# First Whole-Genome Sequence of a *Haemophilus influenzae* Type e Strain Isolated from a Patient with Invasive Disease in Italy

**DOI:** 10.1128/genomeA.00059-17

**Published:** 2017-03-30

**Authors:** Maria Giufrè, Rita Cardines, Marina Cerquetti

**Affiliations:** Department of Infectious Diseases, Istituto Superiore di Sanità, Rome, Italy

## Abstract

In the present era of conjugate vaccines against *Haemophilus influenzae* type b, non-vaccine-preventable strains are of concern. Here, we report the first whole-genome sequence of an invasive *H. influenzae* type e strain. This genomic information will enable further investigations on encapsulated non-type b *H. influenzae* strains.

## GENOME ANNOUNCEMENT

*Haemophilus influenzae* is a Gram-negative bacterium capable of causing respiratory tract infections as well as severe invasive disease. This species is characterized by the presence/absence of a polysaccharide capsule (1). Encapsulated* H. influenzae* expresses one of the six structurally and antigenically distinct capsular polysaccharides (serotypes a to f); nonencapsulated isolates are commonly referred to as nontypeable *H. influenzae* (NTHi). Since the introduction of conjugate vaccines against *H. influenzae* type b (Hib), invasive disease caused by this serotype has drastically decreased (2). However, invasive disease due to *H. influenzae* has not been eliminated, and NTHi and encapsulated non-type b *H. influenzae* strains are nowadays responsible for most invasive infections (3, 4). In Italy, NTHi was found to predominate among invasive isolates, but a number of cases were associated with type e and type f strains (5). In particular, invasive *H. influenzae* type e (Hie) disease has been observed since the beginning of the 2000s in our country and, recently, Hie strains were found to account for the 9% of all invasive *H. influenzae* isolates (5–7). In this study, we determined the whole-genome sequence of an Hie strain isolated from the cerebrospinal fluid from a 66-year-old patient with meningitis in 2014, in Italy. The *H. influenzae* isolate was sent to the reference laboratory of the National Surveillance of Invasive Bacterial Disease at the Istituto Superiore di Sanità (Rome, Italy), where, following genotyping confirmation of the serotype (8), we subjected it to whole-genome sequencing. To our knowledge, no published whole-genomic data are available for Hie.

Genomic DNA was extracted from an overnight culture at 37°C using the NucleoSpin DNA extract kit (Macherey-Nagel, Duren, Germany). Whole-genome sequencing was performed using Illumina MiSeq (250-bp paired-end reads) technology (Illumina, San Diego, CA). The genome sequences were assembled *de novo* using Newbler (9). The final assembly consists of 1,850,604 bp in 140 contigs, with an *N*_50_ of 29,763 bp. The resulting coverage was 310×. Genome annotation was performed using Glimmer3 (10) and the NCBI Prokaryotic Genome Annotation Pipeline (PGAP) (http://www.ncbi.nlm.nih.gov/genome/annotation_prok/), and 1,873 putative protein-coding genes were identified. No plasmid or resistance gene was found by submitting the genome at the Center for Genomic Epidemiology (CGE) server (https://cge.cbs.dtu.dk/services/), using PlasmidFinder and ResFinder, respectively. Multilocus sequence type (MLST) was assigned according to the *H. influenzae* MLST website scheme (http://pubmlst.org/hinfluenzae/) and it was sequence type 18 (ST18). Various putative genes encoding virulence factors were identified, including adherence factors (*hap* and type IV pili), endotoxins (*kdsB* and *lpxK*), lipooligosaccharide (LOS) (*galE* and *lic*), and host immune evasion proteins (*mrsA* and *iga1*).

Detailed analysis of the reported genome, including comparisons with other encapsulated and nonencapsulated *H. influenzae* genomes, is under way. The whole-genome sequence reported here will provide a reference data set for Hie, improving our understanding on the genomic diversity of encapsulated non-type b *H. influenzae* strains.

### Accession number(s).

This whole-genome shotgun project has been deposited at DDBJ/ENA/GenBank under the accession no. MTBH00000000. The version described in this paper is version MTBH01000000.

## References

[1] Pittman M 1931 Variation and type specificity in the bacterial species *Haemophilus influenzae*. J Exp Med 53:471–492. 19869858 10.1084/jem.53.4.471 PMC2131978

[2] Peltola H 2000 Worldwide *Haemophilus influenzae* type b disease at the beginning of the 21st century: global analysis of the disease burden 25 years after the use of the polysaccharide vaccine and a decade after the advent of conjugates. Clin Microbiol Rev 13:302–317. 10756001 10.1128/cmr.13.2.302-317.2000 PMC100154

[3] Ladhani S, Slack MP, Heath PT, von Gottberg A, Chandra M, Ramsay ME, European Union 2010 Invasive *Haemophilus influenzae* disease, Europe, 1996-2006. Emerg Infect Dis 16:455–463. doi:10.3201/eid1603.090290. 20202421 PMC3322004

[4] Langereis JD, de Jonge MI 2015 Invasive disease caused by nontypeable *Haemophilus influenzae*. Emerg Infect Dis 21:1711–1718. doi:10.3201/eid2110.150004. 26407156 PMC4593434

[5] Giufrè M, Cardines R, Caporali MG, Accogli M, D’Ancona F, Cerquetti M 2011 Ten years of Hib vaccination in Italy: prevalence of non-encapsulated *Haemophilus influenzae* among invasive isolates and the possible impact on antibiotic resistance. Vaccine 29:3857–3862. doi:10.1016/j.vaccine.2011.03.059. 21459175

[6] Cerquetti M, Ciofi degli Atti ML, Cardines R, Salmaso S, Renna G, Mastrantonio P, Hi Study Group 2003 Invasive type e *Haemophilus influenzae* disease in Italy. Emerg Infect Dis 9:258–261. 12604001 10.3201/eid0902.020142 PMC2901939

[7] Cerquetti M, Ciofi degli Atti ML, Cardines R, Giufré M, Romano A, Mastrantonio P 2004 *Haemophilus influenzae* serotype e meningitis in an infant. Clin Infect Dis 38:1041. doi:10.1086/382083. 15034842

[8] Falla TJ, Crook DW, Brophy LN, Maskell D, Kroll JS, Moxon ER 1994 PCR for capsular typing of *Haemophilus influenzae*. J Clin Microbiol 32:2382–2386. 7814470 10.1128/jcm.32.10.2382-2386.1994 PMC264070

[9] Myers EW, Sutton GG, Delcher AL, Dew IM, Fasulo DP, Flanigan MJ, Kravitz SA, Mobarry CM, Reinert KH, Remington KA, Anson EL, Bolanos RA, Chou HH, Jordan CM, Halpern AL, Lonardi S, Beasley EM, Brandon RC, Chen L, Dunn PJ, Lai Z, Liang Y, Nusskern DR, Zhan M, Zhang Q, Zheng X, Rubin GM, Adams MD, Venter JC 2000 A whole-genome assembly of *Drosophila*. Science 287:2196–2204. 10731133 10.1126/science.287.5461.2196

[10] Delcher AL, Bratke KA, Powers EC, Salzberg SL 2007 Identifying bacterial genes and endosymbiont DNA with Glimmer. Bioinformatics 23:673–679. doi:10.1093/bioinformatics/btm009. 17237039 PMC2387122

